# Polyphenol-Rich Strawberry Extract Protects Human Dermal Fibroblasts against Hydrogen Peroxide Oxidative Damage and Improves Mitochondrial Functionality 

**DOI:** 10.3390/molecules19067798

**Published:** 2014-06-11

**Authors:** Francesca Giampieri, José M. Alvarez-Suarez, Luca Mazzoni, Tamara Y. Forbes-Hernandez, Massimiliano Gasparrini, Ana M. Gonzàlez-Paramàs, Celestino Santos-Buelga, José L. Quiles, Stefano Bompadre, Bruno Mezzetti, Maurizio Battino

**Affiliations:** 1 Dipartimento di Scienze Agrarie, Alimentari e Ambientali, Università Politecnica delle Marche, Via Ranieri 65, Ancona 60131, Italy; E-Mails: f.giampieri@univpm.it (F.G.); b.mezzetti@univpm.it (B.M.); 2 Dipartimento di Scienze Cliniche Specialistiche ed Odontostomatologiche (DISCO)-Sez. Biochimica, Facoltà di Medicina, Università Politecnica delle Marche, Ancona 60131, Italy; E-Mails: l.mazzoni@univpm.it (L.M.); tamara.forbe@gmail.com (T.Y.F.-H.); m.gasparrini@univpm.it (M.G.); 3 Grupo de Investigación en Polifenoles (GIP-USAL), Faculty of Pharmacy, Salamanca University, Campus Miguel de Unamuno, Salamanca E37007, Spain; E-Mails: paramas@usal.es (A.M.G.-P.); csb@usal.es (C.S.-B.); 4 Department of Physiology, Institute of Nutrition and Food Technology ‘‘José Mataix”, Biomedical Research Centre, University of Granada, Granada 18100, Spain; E-Mail: jlquiles@ugr.es; 5 Dipartimento di Scienze Biomediche e Sanità Pubblica, Facoltà di Medicina, Università Politecnica delle Marche Via Ranieri 65, Ancona 60131, Italy; E-Mail: s.bompadre@univpm.it; 6 Area de Nutrición y Salud, Universidad Internacional Iberoamericana (UNINI), Campeche, C.P.24040, Mexico; 7 Director Centre for Nutrition & Health, Universidad Europea del Atlantico (UEA), Santander 39011, Spain

**Keywords:** strawberry, anthocyanin, ROS, lipid peroxidation, DNA damage, fibroblasts

## Abstract

Strawberry bioactive compounds are widely known to be powerful antioxidants. In this study, the antioxidant and anti-aging activities of a polyphenol-rich strawberry extract were evaluated using human dermal fibroblasts exposed to H_2_O_2_. Firstly, the phenol and flavonoid contents of strawberry extract were studied, as well as the antioxidant capacity. HPLC-DAD analysis was performed to determine the vitamin C and *β*-carotene concentration, while HPLC-DAD/ESI-MS analysis was used for anthocyanin identification. Strawberry extract presented a high antioxidant capacity, and a relevant concentration of vitamins and phenolics. Pelargonidin- and cyanidin-glycosides were the most representative anthocyanin components of the fruits. Fibroblasts incubated with strawberry extract and stressed with H_2_O_2_ showed an increase in cell viability, a smaller intracellular amount of ROS, and a reduction of membrane lipid peroxidation and DNA damage. Strawberry extract was also able to improve mitochondrial functionality, increasing the basal respiration of mitochondria and to promote a regenerative capacity of cells after exposure to pro-oxidant stimuli. These findings confirm that strawberries possess antioxidant properties and provide new insights into the beneficial role of strawberry bioactive compounds on protecting skin from oxidative stress and aging.

## 1. Introduction

There is evidence that oxidative stress, exerting downstream effects such as lipid peroxidation, DNA damage and mitochondrial impairment, may have a causative role in skin disease and aging [1,2]. The skin is the largest organ of the body that creates a self-repairing barrier, protecting the body from the most common potentially harmful physical, environmental, and biological insults. Indeed, there are many factors to which skin is exposed like smoke, microorganisms, or UV radiation, that can induce biological responses, leading to skin damage through the generation of reactive oxygen species (ROS). Human dermal fibroblasts (HuDe) are the main experimental model *in vitro* for studying cellular aging [3,4]; at the same time, some agents such as H_2_O_2, _UV-radiation and lipopolysaccharide are usually used to induce HuDe oxidative damage and premature senescence [5,6]. In recent years several studies have shown that oxidative stress and senescence in human fibroblasts can be counteracted and delayed by certain protective antioxidant compounds [1,4,7]: dietary vitamins and polyphenols are indeed able to ameliorate oxidative skin damage and prevent aging, even if the role of diet in protecting the skin is highly controversial due to the limited amount of scientific data available [8]. For these reasons, in the last few years topical treatment of these compounds has been proposed as a strategic tool in order to provide additional protection from oxidative damage and delay skin aging [8,9].

The strawberry (*Fragaria x ananassa*) is one of the richest natural sources of bioactive compounds, like vitamin C, β-carotene and polyphenolics (phenols, flavonoids, phenolic acids, lignans, and tannins) which express remarkable antioxidant capacities both *in vitro* and *in vivo* [10,11]. Over the last decade, our research has addressed not only the characterization of the nutritional quality of several strawberry genotypes but also the *in vitro* and *in vivo* assessment of the effects of strawberry treatment/consumption on antioxidant status [1,12,13,14,15]. The aim of the present study is to evaluate the *in vitro* effects of a polyphenol-rich strawberry extract in cytoprotection against oxidative stress using H_2_O_2_ as stressors in HuDe cells, since it represents a valid model for studying oxidative stress and senescence on cells. We evaluated the protective effects of strawberry extract on cell viability, ROS concentration, lipid peroxidation and DNA damage. Finally, because mitochondria are the main source of reactive species, which are by-products of cell energy production, within most cells and are the main organelles involved in the aging phenotype, we also assessed the protective effects of strawberry extract on mitochondrial status and functionality. 

## 2. Results and Discussion

### 2.1. Nutrients, Phenolics and Antioxidant Capacity of Strawberry Extracts

According to our previous results [1], the *Sveva* cultivar is a remarkable source of phenols and flavonoids (Table 1); five anthocyanin pigments were detected through HPLC-DAD/ESI-MS analysis, with Pg- and Cy-glycosides being the most representative anthocyanin strawberry components (Table 1). Pg-3-glucoside is the most abundant, with 61.12 mg/100 g FW, followed by Cy-3-glucosides, with a concentration of about 1.63 mg/100 g FW. 

**Table 1:** Nutrient composition, phytochemical content and antioxidant capacity of strawberry extract.

Parameter		Quantification
Vitamin C (mg/g)		0.47 ± 0.04
β-Carotene (µg/100 g)		25 ± 0.02
Total phenolic (mg/g)		2.19 ± 0.09
Total flavonoid (mg/g)		0.70 ± 0.01
Anthocyanins (mg/100 g)		
	Cy-3-glucoside	1.63 ± 0.04
	Pg 3-glucoside	61.12 ± 0.13
	Pg 3-rutinoside	0.19 ± 0.01
	Pg 3-malonylglucoside	0.28 ± 0.02
	Pg 3-acetylglucoside	0.72 ± 0.03
TAC (µmol TE/g)		
	FRAP	14.94 ± 0.39
	TEAC	22.59 ± 0.9

Two important antioxidant compounds present in the strawberry are vitamin C and *β*-carotene. As previously reported by different authors [11,16], they are versatile antioxidants, preventing both singlet oxygen and free radical mediated damage. Based on these findings, we analysed the vitamin C and *β*-carotene content of the extract by HPLC-DAD (Table 1) and found a good content of these compounds (about 47.9 mg/100 g FW and 25 µg/100g FW, respectively), that could contribute to the high antioxidant capacity and free radical scavenging capacity of this cultivar. 

The TAC of fruit extract was quantified by FRAP and TEAC assays (Table 1). *Sveva* extract showed a high TAC value, according to these methods, being 14.94 µmol TE/g and 22.59 µmol TE/g of FW for FRAP and TEAC, respectively. We found that TAC values of the *Sveva* cultivar were similar to those previously reported in other strawberry varieties, and confirmed that this cultivar possesses a high TAC [1].

### 2.2. Cytotoxicity Evaluation

**Figure 1:**
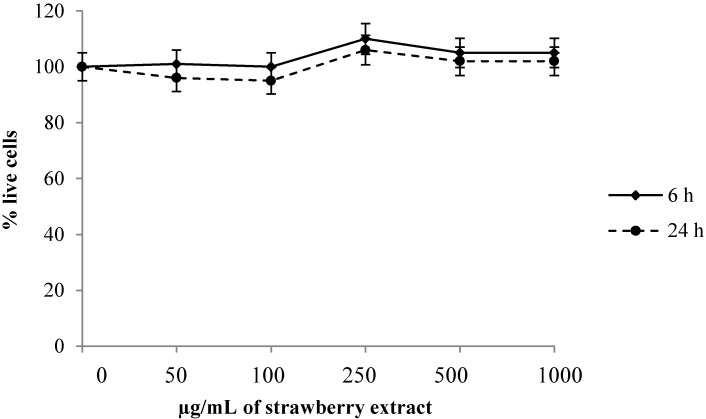
Viability of Human Dermal fibroblast (HuDe) determined by MTT assay after incubation with different concentrations of strawberry extract and at different times.Data are expressed as mean ± SEM for eight replicas (*n* = 8) of three independent experiments.

Firstly, the possible toxic effect of the strawberry extract in relation to its increasing concentration and exposure time was studied (Figure 1). 

**Figure 2:**
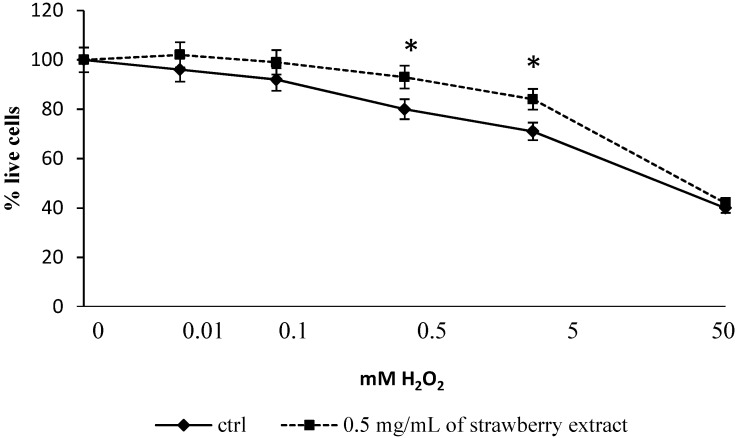
Viability of Human Dermal fibroblast (HuDe) determined by MTT assay after incubation with H_2_O_2_. Control and strawberry pre-treated cells (0.5 mg/mL) were stressed with various concentrations of H_2_O_2_ (0–50 mM). Cells with extract differ significantly from controls for concentrations of H_2_O_2_ between 0.5 and 5 mM. Data are expressed as mean ± SEM for eight replicas (*n* = 8) of three independent experiments. * *p* < 0.05 significantly different from control.

Cell vitality did not vary depending on the strawberry extract concentration or the exposure time, thus no cytotoxic effect was found at the chosen experimental conditions. Therefore, the extract concentration that gave the best results in terms of cell viability and reproducibility (0.5 mg/mL) was selected for all the tests. 

After incubation with H_2_O_2_ for 1 h, only strawberry pre-treated cells did not show a significant decrease in their viability (Figure 2), especially at H_2_O_2 _ concentration of 0.5 and 5 mM, where cell viability remained higher than 80% (*p* ˂ 0.05) compared to control cells. 

**Figure 3:**
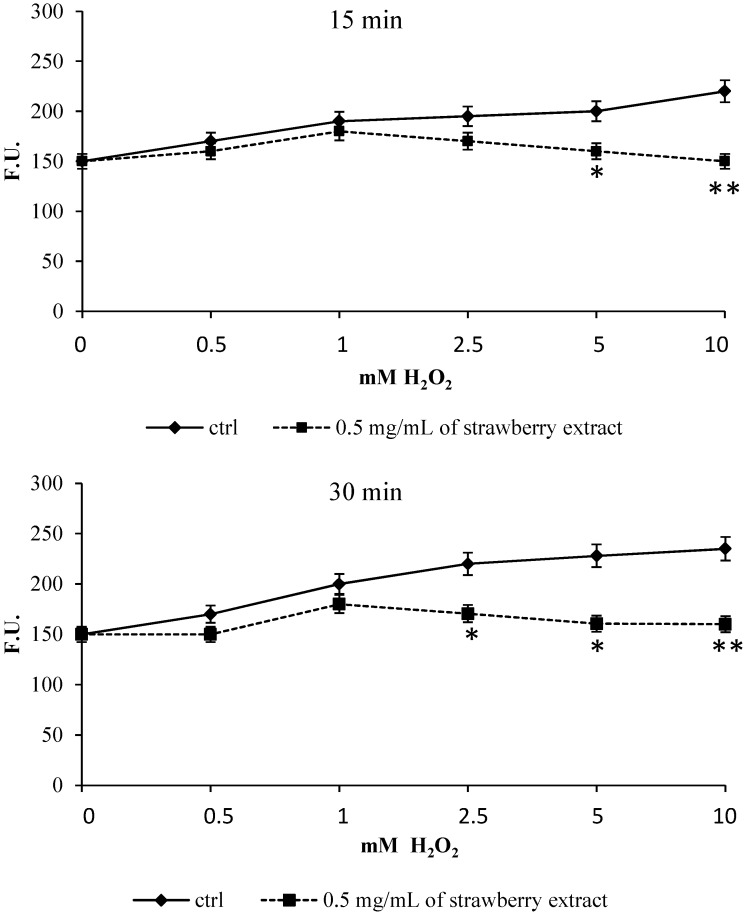
Levels of ROS following H_2_O_2_-induced stress. Control and strawberry extract pre-treated cells were incubated with H_2_DCFDA and then stressed with different concentrations of H_2_O_2_ (0–10 mM). The kinetics of fluorescence was studied for two hours. Variation of fluorescent signal is significant already after 15 min of stress for H_2_O_2_ ≥ 5 mM and after 30 min for concentrations of 2.5 mM. Results are reported as mean DCF fluorescence activity (arbitrary units) obtained from at least three separate experiments (error bars represent SEM). * *p* < 0.05 significantly different from control, ** *p* < 0.01 highly significant compared to control.

### 2.3. Intracellular ROS Production and Lipid Peroxidation

Incubation with strawberry extract was able to decrease intracellular ROS production induced by H_2_O_2_-mediated oxidative damage. Results shown in Figure 3 are expressed as changes in cell fluorescence *versus* oxidant concentrations for different incubation times. After 15 min of incubation with H_2_O_2_ a significant difference of fluorescence was found between control cells and cells incubated with the strawberry extract, especially for H_2_O_2_ concentration ≥ 5 mM (*p* ˂ 0.05) and H_2_O_2_ ≥ 10 mM (*p* ˂ 0.01). After 30 min of incubation, a significant difference (*p* ˂ 0.05) was also found in cells stressed with H_2_O_2_ ≥ 2.5 mM (*p* < 0.05) and these differences remained along all the experimental interval times tested (up to 120 min). 

Part of the protective effects of the strawberry extract may be reasonably attributed to its anthocyanin fraction. In fact, Cy-3-rhamnoglucoside has been found to be very efficient in scavenging free radicals [17] and at the same time anthocyanidins seem to be able to protect human foetal lung fibroblasts from toxicity induced by linoleic acid hydroperoxide: this could be due to the fact that anthocyanins and their glycosidic forms act as antioxidants in lipid environments *in vitro* [18].

**Figure 4:**
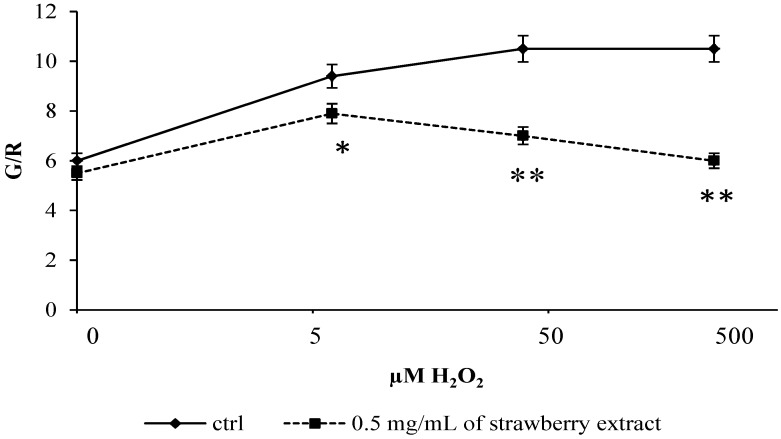
Evaluation of lipid peroxidation after H_2_O_2_ stress. Control and pre-treated cells with extract were incubated with BODIPY for 30 min and stressed with different concentrations of H_2_O_2_ (0–500 μM). The relationship between red and green fluorescence over time decreases significantly for concentrations of 5 μM hydrogen peroxide and highly significantly for concentrations ≥ 50 μM compared to controls. Data are expressed as mean ± SEM for eight replicas (*n* = 8) of three independent experiments. * *p* < 0.05 significantly different from control, ** *p* < 0.01 highly significant.

Cells pre-incubated with *Sveva* also showed a significant decrease (*p* < 0.05) in lipid peroxidation (Figure 4), especially for H_2_O_2_ 5 μM and a highly significant one (*p* < 0.01) for H_2_O_2_ ≥ 50 μM compared to controls. Again, strawberry extract provided protection at cell membrane levels thanks to its anthocyanin that localizes on the polar surfaces of phospholipid bilayers in a region suitable for scavenging aqueous oxygen radicals and lipophilic radicals incorporated into the membranes [17,18]. In accordance with these results and with our findings, we can suppose that the prevention of lipid peroxidation through the scavenging of lipid hydroperoxides could be one of the routes by which anthocyanins can protect cells from oxidative damage.

### 2.4. Mitochondrial Functionality

**Figure 5:**
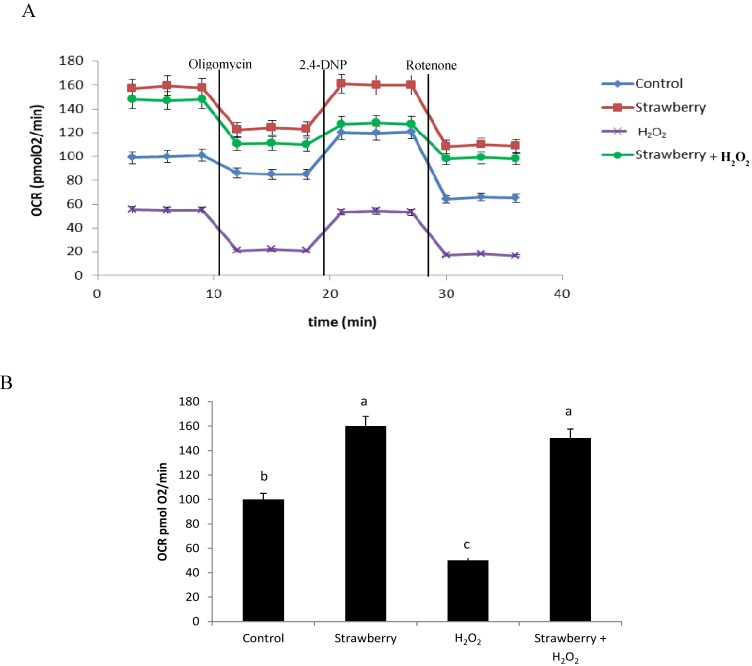
Evaluation of oxygen consumption rate (OCR) in control and in strawberry pre-treated fibroblasts, stressed with H_2_O_2_. OCR was monitored through Seahorse XF-24 Extracellular Flux Analyzer with the sequential injection of Oligomycin (1 µg/mL), 2,4-DNP (100 µM), Rotenone (1 µM) at the indicated time point into each well, after baseline rate measurement (**A**). Basal OCR levels in control, strawberry pre-treated fibroblasts and in cells stressed with H_2_O_2_ with and without pretreatment with the strawberry extract (**B**).

In eukaryotic cells mitochondrial energy metabolism is recognized as the main source of ROS, which can increase because of extracellular oxidative damage. We tested the effect of strawberry extract in protecting mitochondrial bioenergetics against oxidative damage mediated by hydrogen peroxide, which can act as a physiological stressor agent in human dermal fibroblasts. To examine the potential improvement of strawberries on mitochondrial function, the oxygen consumption rate (OCR) was measured in control and pre-treated cells, exposed sequentially to each of three well-defined small modulators of oxidative phoshorylation: oligomycin, 2,4-dinitrophenol (2,4-DNP) and rotenone (Figure 5A). 

The treatment with H_2_O_2_ markedly affected mitochondrial function, reducing the basal OCR values by approximately 1.80 fold compared to control cells, showing the damaging effect of this oxidizing agent on mitochondrial functionality. However, basal OCR was markedly improved (*p* < 0.05) in strawberry-treated cells, highlighting the protection of the extract against H_2_O_2_. The increase of basal OCR in cells after strawberry treatment was approximately 1.59 fold compared to control cells, while pre-treatment with strawberry extract before H_2_O_2_–induced oxidative damage increased basal OCR by approximately 2.70 fold compared to cells treated only with H_2_O_2_. Interestingly, strawberry treatment maintained basal OCR at similar levels in both treatments, demonstrating the possible effect that the bioactive compounds present in the extract may affect, directly or indirectly, mitochondrial functionality (Figure 5B).

Moreover, a similar response was observed in the sequence injections of mitochondrial inhibitors in all experimental groups. In all cases OCR levels were higher in cells pre-treated with strawberry extract, compared to untreated cells. Addition of oligomycin, a substrate that inhibits the ATP synthase activity, caused a significant decrease (*p* < 0.05) of the OCR rate in all samples. The subsequent addition of 2,4-DNP, an ionophore that allows the electron flux without ATP production, increased OCR in all groups and the final addition of rotenone, a substrate that inhibits flux electrons from complex I to Ubiquinone, caused a marked decrease of OCR in all experimental groups.

### 2.5. Inhibition of DNA Damage

**Figure 6:**
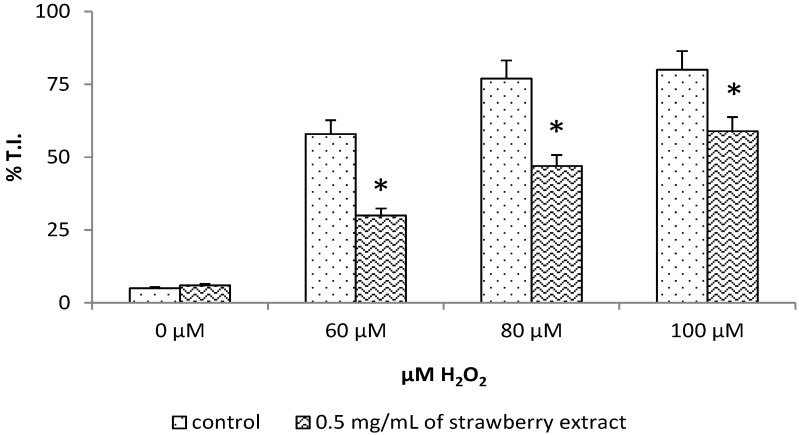
Comet assay after H_2_O_2_ stress. Cells were stressed with H_2_O_2_ for 15 min, processed and analysed for DNA damage. The data are expressed as the percentage of intensity fluorescence in the comet tail (percentage tail intensity) in control and pre-treated cells and relate to three independent experiments performed in triplicate. Statistical analysis was performed with T-test. * *p* < 0.05 significantly different from control.

The results of this study also show that *Sveva* extract protected HuDe cells and prevented DNA damage. Figure 6 shows results expressed as percentage of tail intensity, *i.e.*, intensity of fluorescence in the comet tail which is considered the best parameter for evaluating the extent of cellular DNA damage [1]. DNA damage significantly (*p* < 0.05) decreased in pre-treated cells compared to controls, for concentration of H_2_O_2_ ≥ 60 μM. 

Although anthocyanins are well known to have antioxidant activity [19,20,21], there is very limited evidence about the protective role of these substances against DNA damage. As previously reported by Ghosh *et al.* [22] anthocyanins and phenolic fractions of blackcurrants were more effective in protecting DNA of HL-60 human promyelocytic cells from damage than similar fractions from boysenberries. The phenolic extract of blackcurrants demonstrated the highest protective effect against H_2_O_2_-induced neurotoxicity, oxidative stress and DNA damage. Another study reported that anthocyanin-rich blackberry extract is able to suppress DNA-damaging properties of topoisomerase I and II poisons in colon carcinoma cells. Interestingly, the authors demonstrated that anthocyanidin delphinidin acts as a catalytic topoisomerase inhibitor suppressing the DNA-strand breaking effects of different topoisomerase poisons [23].

**Figure 7:**
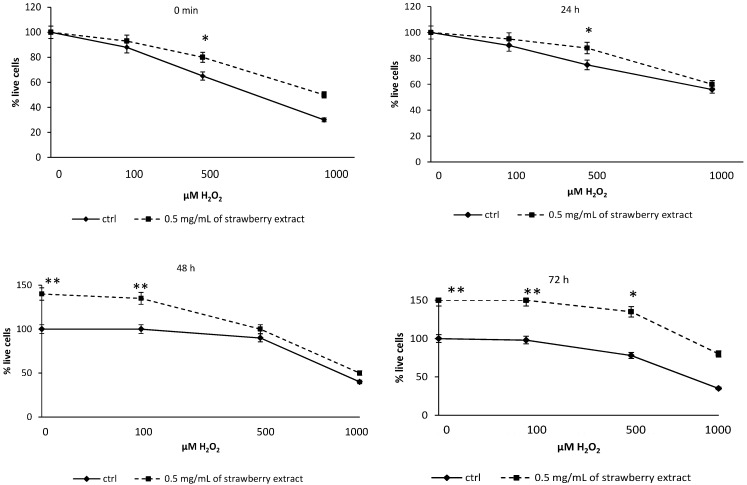
MTT and recovery after stress by H_2_O_2_. Control and pre-incubated cells were stressed with different concentrations of H_2_O_2_ (0.1, 0.5 and 1 mM) and analysed in different times to assess the percentage of vitality. At 0 and 24 h after stress, the difference from controls was significant for concentrations of hydrogen peroxide ≥ 500 mM. After 72 h the two curves differed for all the experimental points. Data are expressed as mean ± SEM for eight replicas (*n* = 8) of three independent experiments. Statistical analysis was performed with the T-test. * *p* < 0.05 significantly different from control, ** *p* < 0.01 highly significantly.

### 2.6. Cell Proliferation and Recovery Assay

With MTT viability assay it was possible to assess whether strawberry extract was also able to influence the recovery of cell viability after prolonged oxidative stress to different H_2_O_2_ concentrations. As demonstrated in tests of viability for control and pre-treated cells, the curve of pretreated cell viability showed a significant difference (*p* < 0.05) with controls for concentrations of H_2_O_2_ ≥ 500 µM. After 24 h (Figure 7) the significance of recovery was recorded only for cells stressed with 500 µM H_2_O_2_. After a further 24 h, the difference between the two curves became highly significant (*p* < 0.01) for ≤ 50 µM H_2_O_2_ and this difference remained after 72 h, when the significance was recorded for all stress points analyzed. In conclusion, evidence of recovery revealed a significant restoring of vitality and cell function after 48 and 72 h from stress induced by H_2_O_2_.

## 3. Experimental

### 3.1. Chemicals

All solvents were HPLC grade. 2,2'-Azinobis(3-ethylbenzothiazolne-6-sulfonic acid) diammonium salt (ABTS), sodium chloride (NaCl), agarose, hydrogen peroxide (H_2_O_2_), ferrous sulphate (FeSO_4_) and all other reagents and solvents were purchased from Sigma-Aldrich Chemie GmbH (Steinheim, Germany). C11-BODIPY was obtained from Molecular Probes (Invitrogen, Milan, Italy) and Minisart filter from PBI International. Essential Modified Eagle Medium (EMEM) and all the other products for cell culturewere purchased from Cambrex (Milan, Italy). 

### 3.2. Strawberry Material and Sample Preparation

Ripe fruits from strawberries of the *Sveva* cultivar were harvested from plants grown in an open experimental field for strawberry breeding and germplasm collection at the Azienda Agraria Didattico Sperimentale ‘‘P. Rosati” in Agugliano (Ancona, Italy). Within 2 h after harvest, whole fruits were stored at −80 °C for further analysis.

For the analysis of phytochemicals, total antioxidant capacity (TAC) and for cell treatment, the hydroalcoholic extract was obtained from 10 g of fruits homogenised for 2 min in 100 mL (1:10 w/v) of extraction solution (80% methanol aqueous solution acidified with 0.1% formic acid) using an Ultraturrax T25 homogeniser (Janke & Kunkel, IKA Labortechnik, Staufen, Germany). After homogenization the extraction was maximised by stirring the suspension for 2 h at 4 °C in the dark. The mixture was then centrifuged at 1200 *xg* for 15 min (twice sequentially), to sediment solids, supernatants were filtered through a 0.45 µm Minisart filter (PBI International), transferred to 5 mL amber glass vials and stored at −80 °C prior to analysis.

For the HPLC-DAD-MS identification and quantification of anthocyanin, extraction and purification was performed as previously described [24] using a C-18 SepPaks Vac 6cc cartridge (Waters, Milan, Italy). Frozen strawberries (50 g) were homogenized in methanol containing 0.1% HCl, kept overnight (~14 h) at 4 °C and later filtered through a Büchner funnel under vacuum. The solid residue was exhaustively washed with methanol and the filtrates obtained were centrifuged (4000 *xg*, 15 min, 20 °C). The resulting extract was washed with *n*-hexane to eliminate liposoluble substances and then an aliquot (4 mL) was deposited onto a C-18 SepPaks Vac 6 cc cartridge (Waters) previously conditioned with methanol and washed with ultrapure water. Sugars and more polar substances were eluded with 15 mL of ultrapure water while anthocyanin pigments were eluted with 5 mL of methanol: 0.1% TFA (95:5). The methanolic extract was concentrated under vacuum in a rotary evaporator and the extract was then collected, brought to 2 mL of volume with ultrapure water and filtered through a 0.45-mm membrane filter (PBI international, Milan, Italy) for HPLC analysis. 

For vitamin C determination, fruits stored at −80 °C were ground to a fine powder using a precooled coffee grinder (IKA A11 basic). Immediately before analysis, 2 mL of ice-cold extraction solution was added to 0.5 g FW frozen powder weighed in 10 mL ice-cold glass tubes, and the mixture sonicated at 4 °C for 15 min in the dark. The extraction solution consisted in MilliQ water containing 5% meta-phosphoric acid and 1 mM EDTA, stirred well prior to use, sonicated until dissolved and stored in the refrigerator. After the ultra-sound assisted extraction, cell walls and proteins were precipitated by centrifugation at 2500 rpm for 10 min at 4 °C, the surnatant was filtered through a 0.2 μm PTFE filter into 1.8 mL HPLC vials, and immediately analysed. 

For *β*-carotene analysis, 30 mL acetone were added to 5 g of freeze-dried fruits powder, sonicated for 15 min, stirring for 1 h in the dark at room temperature and centrifuged twice at 9500 *×g* at 4 °C for 15 min. The extract was combined and brought to final volume of 100 mL, and 50 mL of methanolic KOH (10%) was added for saponification at room temperature overnight. The extract was then transferred to 100 mL petroleum ether and the organic layer was dried. The dried residue was dissolved in hexane and filtered through a 0.45 µm membrane filter for HPLC analysis. 

### 3.3. Measurement of Total Phenolic Content

The total phenolic content of the hydrophilic extracts was determined using the Folin-Ciocalteu colorimetric method, as modified by Slinkard and Singleton [25]. Briefly, 100 μL of alternatively blank (milliQ water), water diluted strawberry extracts or gallic acid standard solutions (1/10) were added to 500 μL of Folin-Ciocalteau reagent previously water diluted (1/10) and kept at 4 °C, in the dark. The mixture was incubated for 1 to 8 min at room temperature, then 400 μL of 0.7 M sodium carbonate (Na_2_CO_3_) was added and the mixture vortexed well. The solution was incubated for 2 h at room temperature (~23 °C), in the dark, then the specific absorbance at 760 nm was read. A methanol–water (80:20, v/v) solution of 6 mM gallic acid (GA) was prepared, and stored at 4 °C for at maximum one week. Serial standard dilutions (0.5–3.0 mM) were daily prepared from the stock solution, for quantifications. Calibration was obtained by plotting the known GA concentrations *versus* the corresponding absorbance760, and final results were expressed as milligrams of gallic acid equivalents per gram of fresh weight of strawberry [mg GAEq/g FW] (mean value of eight technical replicates) ± SEM.

### 3.4. Measurement of Total Flavonoid Content

Total flavonoid content was determined by using a colorimetric method described previously [26]. Briefly, 250 μL of alternatively blank (water), strawberry hydrophylic extract or (+)-Catechin standard solution was mixed to 1.25 mL of MilliQ water in a test tube, following by addition of 75 μL of a 5% sodium nitrate (NaNO_2_) solution. After 6 min, 150 μL of a 10% aluminium chloride hexahydrate (AlCl_3_·6H_2_O) solution was added to the mixture, and allowed to stand for 5 min. Then, 500 μL 1M sodium hydroxide (NaOH) were added, the mixture was brought to 2.5 mL with MilliQ water and mixed well, and the absorbance was immediately read at 510 nm against blank. For quantitative results, from a methanol–water (80:20, v/v) stock solution of (+)-catechin (1 mg/mL), serial standard dilutions were prepared (0.0125–0.1 mg/mL), and their known concentrations *versus* the corresponding absorbance were plotted. Results are expressed as mg of catechin equivalents per gram of fresh weight of strawberry [mg CEq/g FW] (mean value of eight technical replicates) ± SEM.

### 3.5. Determination of Vitamin C and β-carotene

Vitamin C was measured as described by Helsper and co-workers [27]. Strawberry extracts were subjected to HPLC analysis immediately after the extraction procedure. The HPLC system comprised a Waters 600 controller, a Waters 996 Photodiode array (PDA) detector set at absorbances of 262 and 244 nm, and a column incubator at 30 °C. The HPLC column used was a YMC Pack Pro 150x4.6 mm. The elution was isocratic with 50 mM potassium phosphate (KH_2_PO_4_) in MQ water, leading to pH 3.2 (below the pKa of the ascorbic acid) by adding orthophosphoric acid, and analysis consisted in a 10 minute run, after which the column was cleaned with 50% acetonitrile. Vitamin C eluted at RT ≈ 5.3 min. Quantification of the vitamin C content was carried out through a calibration curve prepared by running standard concentrations of vitamin C similarly prepared to the extracts, and measured in duplicate at the beginning and end of the analysis. Results are expressed as mg vitamin C per gram of fresh weight of strawberry [mg vit C/g FW] (mean value of three technical replicates ± SEM).

β-Carotene content was determined with an HPLC method previously described by Zanatta *et al.* [28]. Samples were injected in the HPLC system with detector set at 445 nm at 30 °C. The analytical column used was a Supelcosil™ LC-18 (150 × 4.6 mm) and the mobile phase was acetonitrile–methanol–ethyl acetate (88:10:2 v/v) in isocratic gradient flow at a rate of 1.0 mL/min. For quantification a *β*-carotene standard was used and results are expressed as µg β-carotene per 100 g of FW.

### 3.6. HPLC-ESI-MS Characterization of Strawberry Extract

HPLC-DAD/ESI-MS analysis was performed using a Hewlett-Packard 1100 series liquid chromatograph (Agilent Technologies, Waldbronn, Germany) coupled to an HP ChemStation data-processing station [24]. The stationary phase used was a 5 µm AQUA^®^ C 18 150 mm × 4.6 mm column (Phenomenex, Torrance, CA) thermostatted at 35 °C and the mobile phase was: (A) 0.1% trifluoroacetic acid in water, and (B) HPLC-grade acetonitrile, using the following gradient: isocratic 10% B for 5 min, 10%–15% B over 15 min, isocratic 15% B for 5 min, 15%–18% B over 5 min, and 18%–35% B over 20 min, with a flow rate of 0.5 mL min^−1^. Double on-line detection was performed using a diode array spectrophotometer (DAD) at 520 nm as the selected wavelength, and a mass spectrometer (MS) connected to the HPLC system via the DAD cell outlet. 

The mass spectrometer (Finnigan LCQ, San Jose, CA, USA) was equipped with an ESI source and an ion trap mass analyzer, controlled by LCQ Xcalibur software. Nitrogen was used as both auxiliary and sheath gas at flow rates of 6 and 1.2 L min^−1^, respectively. The capillary voltage was 4 V and the capillary temperature 195 °C. Spectra were recorded in positive ion mode between *m/z* 150 and 1500. The MS detector was programmed to perform a series of three consecutive scans: a full scan, a zoom scan of the most abundant ion in the first scan and an MS–MS scan of the most abundant ion, using normalized collision energy of 45%. Anthocyanins were identified and quantified from the areas of chromatographic peaks by comparison with calibration curves obtained with external standards of Cyanidin-3-glucoside (Cy-3-gluc) and of Pelargonidin 3-glucoside (Pg-3-gluc). 

### 3.7. Total Antioxidant Capacity

The TAC of the strawberry extract was determined by the Trolox Equivalent Antioxidant Capacity (TEAC) and the Ferric Reducing Antioxidant Power (FRAP) assays. The TEAC assay was carried out according to the modified method of Re and co-workers [29] and combined to a flow injection analysis (FIA) system [30]. The strawberry extract (10 µL) was injected into a serpentine-knotted reaction coil and allowed to react with the ABTS+ solution pumped at a flow of 1.2 mL/min into the coil. The extent of decolorization, expressed as percentage of inhibition of absorbance, is then plotted as a function of concentrations of the antioxidant capacity in the sample. TEAC results are expressed as micromoles of Trolox equivalents per gram of FW of strawberry. Data are reported as a mean value ± SEM for four measurements.

The FRAP assay was carried out according to the protocol proposed by Deighton and co-workers [31]. The FRAP reagent solution was prepared daily immediately prior to procedure, by combining ten volumes of sodium acetate (300 mM, pH 3.6) with one volume of TPTZ (10 mM in HCl 40 mM) and one volume of ferric chloride (20 mM) aqueous solutions. Briefly, 100 μL of alternatively blank, Trolox standard or 10-fold milliQ water diluted strawberry extract were added to 900 μL FRAP reagent. The mixture was then quickly vortexed for 15 s (starting the timer immediately) and allowed to stand for 4 min. After exactly 4 min, the absorbance of the solution was read at 593 nm (model DU644 spectrophotometer, Beckman, Fullerton, CA, USA) against blank. Trolox aqueous dilutions were used for calibration. Each sample was analyzed in eight replicates and FRAP results were expressed as micromoles of Trolox equivalents per gram of FW of strawberry. Data are reported as a mean value ± SEM for four measurements.

### 3.8. Culture of HuDe Cell Line and Cell Treatment

Primary Human Dermal Fibroblasts (HuDe) isolated from adult skin were purchased from the Central Laboratory of Istituto Zooprofilattico Sperimentale (Brescia, Italy). Cells were cultured in EMEM (Cambrex, Milan, Italy) supplemented with 10% foetal bovine serum (FBS), 2 mM glutamine and antibiotics (100 IU/mL penicillin and 100 μg/mL streptomycin), at 37 °C in a humidified atmosphere with 5% CO_2_. 

Strawberry extract was concentrated under vacuum to eliminate total ethanol and resuspended in EMEM to achieve a final concentration of 0.500 mg/mL, reported in our previous work [1]. Cells were incubated for 24 h with *Sveva* extract. After incubation and prior to stress induction the cells were washed twice with PBS, to prevent direct extracellular interactions between the extract compounds and H_2_O_2_, and then exposed to the stressor added to the culture medium at different concentrations and time, according to previously cytotoxicity studies conducted for each test. Control cells were incubated only in EMEM.

### 3.9. Cell Viability Assay

Cell viability was determined using the MTT assay [32]. Control and pre-incubated cells were stressed with different concentrations of H_2_O_2_ (0–50 mM) for 2 h. After incubation, fibroblasts were washed twice with PBS and incubated with a salt solution of MTT at a concentration of 0.5 mg/mL for 2 h at 37 °C. This assay is based on the reduction of the tetrazolium salt, 3-(4,5-dimethylthiazol-2-yl)-2,5-diphenytetrazolium bromide, by intracellular dehydrogenases of viable living cells, leading to the formation of purple formazan crystals. The medium was then removed and the crystals were dissolved in DMSO. Optical density was read at 550 nm using a microplate reader (Synergy HT, Biotek, Winooski, VT, USA). Cell viability was expressed as a percentage of live cells compared to unexposed control. The data reported represent average values from at least three independent experiments.

### 3.10. Evaluation of Intracellular ROS

After pre-incubation with *Sveva* extract cells were incubated with different concentrations of H_2_O_2 _(0–10 mM) and the kinetic production of intracellular ROS was followed for two hours, with readings taking place every 15 min [33]. After treatment with the stressor, cells were washed twice with PBS and incubated with a 20 µM solution of DCFH-DA for 30 min, at 37 °C. Intracellular ROS production was determined kinetically by the change of fluorescence signal (excitation and emission wavelengths: 488 nm and 510 nm, respectively) using a microplate reader (Synergy HT). 

### 3.11. Evaluation of Membrane Lipid Peroxidation

Lipid peroxidation was estimated using the C11-BODIPY, a red fluorescent fatty acid probe used for indexing lipid peroxidation and antioxidant efficacy in living cells [34]. Cells were incubated with 5 μM C11-BODIPY for 30 min at 37 °C, and after washing with PBS were stressed with various concentrations of H_2_O_2_ (0–500 μM). Oxidant action was evaluated for two hours in relation to changes in the relationship between the two fluorescences (excitation wavelength at 485 nm and a double emission wavelength at 528 and 590 nm) using a fluorescence microplate reader (Synergy HT). The extent of lipid peroxidation was quantified as a percentage of red fluorescence increase in pre-treated cells compared to the control.

### 3.12. Evaluation of Mitochondria Respiration

Oxygen consumption rate (OCR) in fibroblasts was measured in real-time using a XF-24 Extracellular Flux Analyzer (Seahorse Bioscience, Billerica, MA, USA) [35]. Cells (2 *×* 10^4^ cells/well) were seeded in the XF-24 plate and incubated for 24 h with the strawberry extract (0.50 mg/mL). After pre-incubation, cells were exposed to H_2_O_2_ (5 mM) for 1 h, according to studies previously conducted to determine the concentration of H_2_O_2_ and time of exposition in order to obtain a reduction of the mitochondrial respiratory capacity of approximately 70% compared to control cells. The medium was then replaced with 450 μL of XF-24 running media and the plate was incubated at 37 °C for 60 min in the XF Prep Station incubator (Seahorse Bioscience). The plate was transferred to the XF24 Extracellular Flux Analyzer and after the measurement of basal OCR a profiling of mitochondrial function was performed by sequential injection of three compounds that affect bioenergetics, as follows: 55 μL of Oligomycin (final concentration 1 µg/mL) at injection in port A, 61 μL of 2,4-dinitrophenol (2,4-DNP) (final concentration 100 μM) at injection in port B, 68 μL of rotenone/Antimycin A (final concentration 1 μM, respectively) at injection in port C. The readings were performed three times for each inhibitor with three min given for each reading and data were expressed as *p*mol of O_2_ consumed per minute.

### 3.13. Evaluation of DNA Damage

Comet assay was performed as described by Singh *et al.* [36] with some modifications [37]. Briefly, cells cultured in 12-well plates were washed twice with PBS and stressed with different concentrations of H_2_O_2_ (0–100 μM). PBS was then removed; cells were detached by trypsinization and resuspended in EMEM. Cells were centrifuged for 15 min at 1,000 *×g* at 4 °C and aliquots containing ~50,000 cells were resuspended in 110 μL of 1% low melting agarose. Then 50 μL of these were placed on a 1% normal melting agarose precoated and dried microscope slide and immediately spread by covering with a coverslip, then the microgel on the slides was allowed to solidify at 4 °C. The cover glasses were removed and the slides were immersed in ice-cold, freshly prepared lysis solution (2.5 M NaCl, 100 mM Na_2_EDTA, 10 mM Tris-HCl, 1% Triton X-100, and 10% DMSO, adjusted to pH 10) for 15 h, at 4 °C in the dark. After removing the slides from the lysis solution these were submerged in an alkaline buffer containing 1 mM NaOH, pH 13, for 20 min in a horizontal electrophoresis box and electrophoresed for 20 min at 1 V/cm at room temperature. After electrophoresis, the slides were washed gently with 0.4 M M Tris-HCl buffer (pH 7.5) to neutralize the excess alkali and to remove detergents. Successively the slides were dehydrated in 75% methanol for 5 min and dried at 60 °C for 15 min and then stained by adding 100 μL of ethidium bromide (20 mg/mL). The analysis was performed using a 20X objective on a fluorescence microscope (Nikon Eclipse E600, Nikon Corporation, Tokyo, Japan) using FITC filters (490 nm excitation/520 nm emissions) and the comet images were analyzed using a CCD camera connected to HP computer. For each sample, 50 comets on two different slides (*i.e.*, 100 comets/sample) were evaluated in each experiment. DNA damage on the slides was quantified by determining the fluorescence intensity in the tail (% tail intensity). 150 randomly selected cells per slide were scored for each sample. 

### 3.14. Cell Proliferation Assay

To evaluate the effect of *Sveva* extract in the recovery of pre-treated cells after prolonged oxidative stress following exposure to H_2_O_2_ (0.1, 0.5 and 1 mM) for 1 h, cells at time 0 were immediately tested as described above with the MTT assay [32]. The remaining cells were incubated with fresh EMEM at 37 °C for 24, 48 and 72 h before being analyzed by the same assay viability.

### 3.15. Statistical Analysis

All results are expressed as means ± SEM. Statistical analysis was performed using the one-way ANOVA and Turkey’s Post Hoc Test; *p* ≤ 0.05 was considered as significant and *p* < 0.01 highly significant.

## 4. Conclusions

Our results report that the *Sveva* strawberry cultivar has a high antioxidant capacity, as well as an important anthocyanin and vitamin content, which results in a protective effect on skin cells against damage induced by oxidative stress. Indeed, strawberry extract was effective in decreasing intracellular ROS concentration and in protecting lipid, DNA and mitochondrial functionality from the damage induced by free radicals. 

Overall, the present study represents an interesting starting point for future investigations. Further studies will be of particular interest, in order to confirm our findings, to explore the direct and indirect antioxidant mechanisms underlying the beneficial effects of strawberry treatment, and to further investigate the role of specific classes of compounds in explaining the reported bioactivities.

Certainly, the authors are aware of the limitations of the study. Firstly, the absence of the key genes expression analysis, involved in antioxidant responses, is an important drawback of the study, since it could have been useful to further corroborate the role of strawberry extract on the results obtained. Another limit of the study is the lack of measurement of DNA damage and cell cycle of key markers involved in cell proliferation by western blot, since it could be essential to examine the mechanisms thorough which strawberry extract contributes to attenuate cytotoxicity induced by hydrogen peroxide. Moreover, more in-depth analysis would have added important data to define the *in vitro* protective effects of strawberry bioactive compounds in different subcellular districts in the recovery from oxidative insults and to verify the bioavailability of these molecules in skin cells after dietary intake or topical application, in order that strawberries can be used also as a natural antioxidant for preventing skin aging and diseases. 

## Supplementary Materials

Supplementary File 1
