# Supplementary material for: Polyphenol-Rich Strawberry Extract Protects Human Dermal Fibroblasts against Hydrogen Peroxide Oxidative Damage and Improves Mitochondrial Functionality
Source: Molecules. 2014 Jun 11;19(6):7798–816. doi: 10.3390/molecules19067798 (PMC6270910; doi:10.3390/molecules19067798)

Supplemental Material

Selection of Comet assay images after H_2O_2 stress. Cells were incubated for 24 h with Sveva extract (0.5 mg/mL). After incubation cells were stressed with different concentrations of H_2O_2 for 15 minutes, processed and analysed for DNA damage.

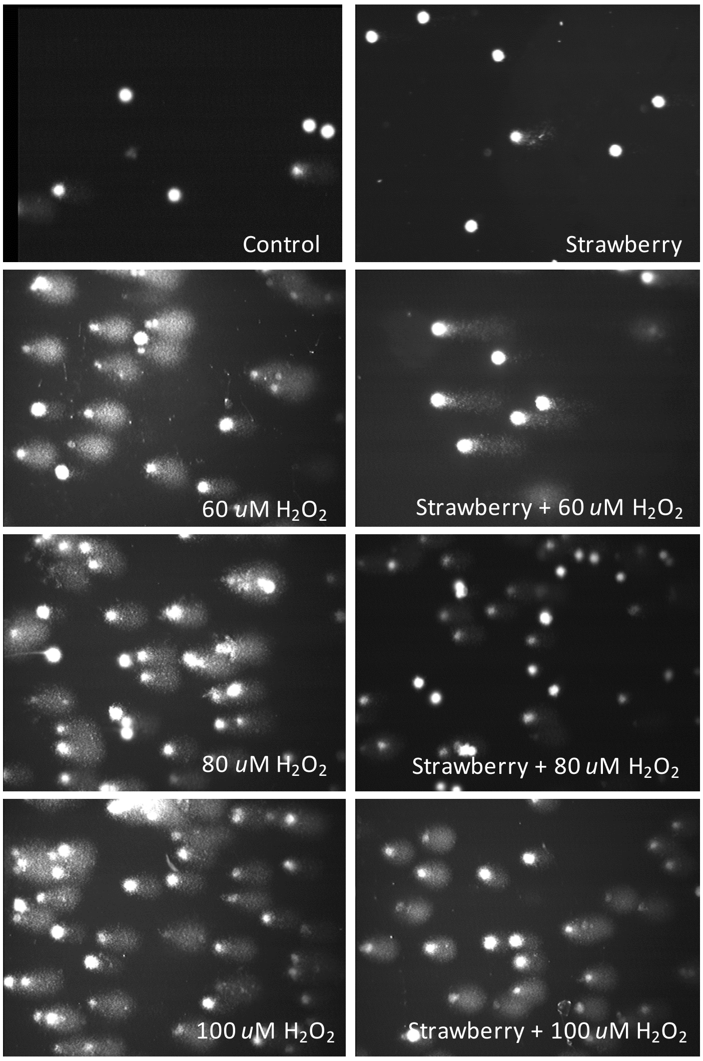

Supplement: Supplementary File 1 [file molecules-19-07798-s001.pdf]
